# Editorial: What does it take to cure the brain? Studies toward genes, proteins, processes, and rehabilitation

**DOI:** 10.3389/fnins.2023.1252955

**Published:** 2023-07-18

**Authors:** Xinhua Shu, Nunzio Denora, Valentino Laquintana

**Affiliations:** ^1^Pu Ai Medical School, Shaoyang University, Shaoyang, Hunan, China; ^2^Department of Biological and Biomedical Sciences, Glasgow Caledonian University, Glasgow, United Kingdom; ^3^Department of Vision Science, Glasgow Caledonian University, Glasgow, United Kingdom; ^4^Department of Pharmacy—Pharmaceutical Sciences, University of Bari “Aldo Moro”, Bari, Italy

**Keywords:** brain dysfunction, brain disorders, aging, Alzheimer's disease, stroke, microglia, treatment

The brain is the most complex organ in the human body, responsible for – among other things –physical and intellectual activity, sensory processing, visceral regulation, hunger and temperature control (Raichle, 2010). Brain dysfunction is characterized by the inability to respond to environmental demands and can be caused by developmental deficit, metabolic abnormality, infection, trauma and aging (Broyd et al., 2009; Moreno-De-Luca et al., 2013). It is associated with a wide range of neural disorders, such as autism, sepsis, stroke, Alzheimer's disease (AD) and depression. Most brain disorders are complex diseases, associated with environmental and genetics factors, and involving complicated signal processing. New technologies, such as transcriptomics, metabolomics and microbiome analysis, have been widely used to elucidate the mechanisms underlying brain pathology. Development of novel treatment for brain disorders requires counteracting multiple targets/signaling pathways, such as infection, oxidative damage, inflammation and neural cell death and regeneration. Currently immunotherapy, cell therapy and gene therapy have shown some benefits to patients with brain disorders (Khan et al., 2020). This Research Topic aims to explore original research and review papers concerned with the elucidation of complicated disease mechanisms and the development of therapeutic strategies for treating brain disorders.

AD is the most common cause of dementia and has two types: early-onset AD and late-onset AD. Early-onset AD is inherited and caused by mutations in *amyloid precursor protein* (*APP*), *presenilin-1* (*PSEN1*) and *presenilin-2* (*PSEN2*) genes. Late-onset AD is a complex disease with involvement of environmental and genetic factors. The clinical features of AD are loss of short-term memory, weakened judgement, impaired learning and visuospatial perception and decreased executive functions. The cardinal features of AD pathology are the accumulation of extracellular amyloid β-protein (Aβ) deposits and intracellular aggregations of hyperphosphorylated tau protein (forming neurofibrillary tangles, NFT). There is a good deal of evidence to demonstrate that both Aβ plaques and NFT are neurotoxic and trigger oxidative damage and inflammation, leading to neural death in the brain, with subsequent cognitive impairment. Microglia are brain-resident immune cells and play an important role in the maintenance of a healthy environment for brain function. Chronic microglia activation contributes to AD pathology by producing proinflammatory factors, spreading Tau aggregates, promoting neurotoxic astrocytes and injuring neurons (Sarlus and Heneka, 2017). Consequently, anti-inflammation therapeutic strategies may benefit AD patients. Anwar et al. investigated the therapeutic effects of primary healthy microglia (PHM) and carbenoxolone (CBX, a common gap junction blocker with anti-inflammatory property) in an AD rat model. They found that intracerebroventricular treatment with PHM or CBX attenuated lipopolysaccharide (LPS)-induced cognitive deficits. PHM and CBX administration also significantly reduced the levels of Aβ and Tau proteins and suppressed expression of glial fibrillary acidic protein (GFAP, a biomarker for astrocytes) in brain and spinal cord of LPS-treated rats. Further, the authors showed that PHM and CBX treatment alleviated LPS-induced neural apoptosis in the cerebral cortex, hippocampus and spinal cord (Anwar et al.). These results suggest that OPHM and CBX have therapeutic potential for AD patients.

Stroke is the most common cerebrovascular disease and a prominent cause of death and long-term disability globally, affecting approximately 13.7 million individuals per year. Ischaemic stroke is the major type of stroke, accounting for over 70% of all stroke cases. Most ischaemic stroke is sporadic, associated with environmental and genetic risk factors (Campbell et al., 2019). Its major clinical feature is arterial occlusion, resulting in inadequate blood supply to cerebral tissue and neural death. Microglia also play a critical role in the pathogenesis of and recovery from ischemic stroke, being injurious by secreting proinflammatory cytokines (M1 form) or beneficial by promoting neuronal recovery (M2 form), depending on microglial states. Remodeling microglial status might therefore be an effective therapeutic strategy for ischemic stroke. Matsumoto et al. treated an ischemic stroke rat model induced by transient middle cerebral artery occlusion (MCAO) with a mixture of granulocyte-macrophage colony-stimulating factor (GM-CSF) and interleukin-3 (IL-3), which can activate microglia and protect against brain injury. The authors found that the cytokine treatment alleviated ischemic injury by reducing infarction and reversing MCAO-caused behavioral deficits. The treatment also stimulated expression of anti-apoptotic factor, Bcl-xL, in neuronal cells, suggesting possible inhibition of neural apoptosis. The authors also noticed that the cytokine treatment supressed expression of proinflammatory cytokines (IL-1β and TNFα) in microglia and facilitated microglial polarization from proinflammatory toward tissue-repairing status. Matrix metalloproteinase-12 (MMP-12) is predominantly produced by macrophages/microglia and can stimulate TNFα production in microglia and boost the production of other proinflammatory mediators. Chelluboina et al. (2015) reported that MMP-12 is significantly upregulated in both acute and chronic ischemic brains of MCAO rats and that knockdown of MMP-12 via small hairpin RNAs (shRNA)-mediated silencing attenuates ischemic brain damage. The same group further examined the optimal timing of MMP-12 shRNA treatment and any gender-dependent efficacy of MMP-12 shRNA treatment. The authors found that immediate treatment after reperfusion was more effective in neurological recovery and that the treatment was effective in both male and female rats, though in females was less apparent. The authors also found that the effectiveness of a single-dose treatment was similar to that of multiple-dose treatment. The authors concluded that suppression of MMP-12 might offer therapeutic potential for patients with ischemic stroke (Challa et al.).

Hypoxic-ischaemic encephalopathy (HIE) is the leading cause of brain developmental injury, affecting approximately 0.2–0.3% of live births. Currently, therapeutic hypothermia is the only available treatment for HIE, increasing survival and decreasing disability rate after HIE (McAdams and Juul, 2016). Alternative therapeutic approaches, such as targeting oxidative damage and inflammation, are being developed. Cannabinoids, originating from cannabis plants, have anti-inflammatory and anti-excitotoxic capacity and show neuroprotective effects in HIE and brain traumatic injury (Xiao et al.). Xiao et al. discussed the main cannabinoid compounds (9-tetrahydrocannabinol and its metabolite, cannabidiol), the endocannabinoid system, endocannabinoid metabolism, and endocannabinoid receptors and related signaling pathways. The authors also considered the role of the cannabinoid and endocannabinoid system in neurodevelopment and gave examples of protection by cannabidiol and cannabinoid agonist against neural injury and behavioral deficits in large HIE models and in rodent stroke models. The authors suggested that the cannabinoid and endocannabinoid system is a therapeutic target for HIE (Xiao et al.).

## Conclusion

In conclusion, this Research Topic collected papers relating to acute brain disorders (e.g., ischemic injury) and chronic brain disorders (e.g., Alzheimer's disease) and provided emerging evidence for treatment of such brain conditions. This Research Topic will contribute to further understanding of the pathological mechanisms of brain disorders and the development of novel therapeutic strategies for patients with brain disorders.

## Author contributions

XS wrote the editorial. ND and VL read the editorial. All authors contributed to the article and approved the submitted version.
